# Comparison of morning versus afternoon cecal intubation rates

**DOI:** 10.1186/1471-230X-7-19

**Published:** 2007-06-08

**Authors:** Christopher D Wells, Russell I Heigh, Virender K Sharma, Michael D Crowell, Suryakanth R Gurudu, Jonathan A Leighton, Nora Mattek, David E Fleischer

**Affiliations:** 1 Division of Gastroenterology & Hepatology, Division of Gastroenterology & Hepatology, Mayo Clinic, 13400 East Shea Boulevard, Scottsdale, AZ 85259, USA; 2 Research Data Analyst, Oregon Health and Science University, Portland, OR 97239, USA

## Abstract

**Background:**

Many factors impacting cecal intubation rates have been examined in detail; however, little information exists regarding the effect of the timing of the procedure. We sought to examine any difference in cecal intubation rates between morning and afternoon colonoscopies and identify factors contributing to a discrepancy.

**Methods:**

Retrospective, single-center study comparing cecal intubation rates for colonoscopies performed in the morning (begun prior to 12 noon) and colonoscopies performed in the afternoon (begun after 12 noon) over an approximately 12 month period. Univariate and multivariate analyses were performed evaluating patient demographics, procedure indication(s), endoscopist, bowel preparation type and quality, and participation by a gastroenterology fellow.

**Results:**

6087 colonoscopies were evaluated in this study. Colonoscopies (n = 3729) performed in the morning were compared to colonoscopies performed in the afternoon (n = 2358). The crude completion rate to the cecum was 95.0% in the morning group while the completion rate to the cecum was 93.6% of the afternoon exams (p = 0.02). The morning colonoscopies had better bowel preparation quality (p < 0.001). The multivariate analyses demonstrated that gender, age, and bowel preparation quality impacted completion rates. After correcting for these factors, there was no significant difference in completion rates in the morning versus afternoon.

**Conclusion:**

Uncorrected cecal intubation rates were lower in the afternoon compared to the morning in outpatients undergoing colonoscopy. Bowel preparation quality was worse in the afternoon compared with the morning. Efforts at improving afternoon bowel preparation may improve the outcome of afternoon colonoscopies.

## Background

Examination of the entire colon is necessary to detect and remove as many polyps as possible to maximize the effectiveness of colorectal cancer screening. [1] A number of factors such as gender, age, prior hysterectomy, body mass index and endoscopist's experience have been identified in the literature as impacting cecal intubation rates. [2-10] Additionally, age, gender, bowel preparation quality, history of constipation, body mass index and endoscopist's experience have been reported to affect procedure duration. [11,12] Cecal intubation rates have been used as a one measure of quality of colonoscopic examination. [13] While any single measure of quality is imperfect, it is generally accepted that cecal intubation rates for both diagnostic and screening colonoscopies should be >90% for individuals and institutions. [13-15] However, this target is not always achieved. [1,15,16] While many of these factors have been studied in depth, the timing of colonoscopy (ie morning versus afternoon) has not been studied in detail. Sanaka, et al, recently reported a lower cecal intubation rate in a retrospective study of 2087 colonoscopies comparing morning and afternoon colonoscopies. They reported a statistically significant difference in cecal intubation rates between morning and afternoon exams after excluding cases limited by poor bowel preparation. [17] The aim of our study was to evaluate the relationship of cecal intubation rates between colonoscopies performed in the morning and afternoon and identify factors contributing to a difference in between the groups using multiple logistic regression analysis.

## Methods

The Internal Review Board at our institution (Mayo Clinic, Scottsdale, AZ) approved the study. All endoscopies performed at our institution are reported in the Clinical Outcomes Research Initiative (CORI) database. Using the CORI database, we identified all outpatient colonoscopies between January 26, 2004 and January 13, 2005. The cecal intubation rates were compared for exams that began prior to 12:00 noon (AM Group) with those begun after 12:00 noon (PM Group). Only colonoscopies that were intended to reach the cecum were included. Each endoscopist determined if the cecum was successfully intubated at the time of the colonoscopy. The cecum was identified by the presence of the appendiceal orifice and the ileocecal valve. If the cecum was not reached, then the colonoscopy was deemed incomplete.

Data on patient age, patient gender, endoscopist, indication(s), type of bowel preparation, quality of bowel preparation and cecal intubation rate was extracted and analyzed. The CORI database requires the assignment of bowel preparation quality in the report as follows: "excellent," "good," "fair, adequate exam," "fair, exam compromised," or "poor." Any examinations lacking appropriate documentation of bowel preparation quality were excluded. Bowel preparations were defined as acceptable ("excellent," "good," or "fair, adequate exam") or unacceptable ("fair, exam compromised," or "poor"). All patients during this time period had been instructed by nursing staff and/or printed literature to complete their bowel preparation the evening prior to the examination. The patients at our institution are instructed to adhere to a clear liquid diet the day prior to the examination and to assume "nothing per mouth" status after midnight. The bowel preparation consists of four liters of polyethylene glycol (PEG) electrolyte solution. Over 95% of patients receive this bowel preparation at our institution. A small minority receives other bowel preparations including phopha soda bowel preparations.

Cecal intubation rates for the AM and PM groups were compared using the Chi-square statistic and 95% confidence intervals. Group comparisons for patient demographics, involvement of gastroenterology fellow, indication(s), type of bowel preparation and quality of bowel preparation were compared using the Chi-square statistic and 95% confidence intervals or the Student's t-test as appropriate for the distribution of each variable. All endoscopists performing at least 50 colonoscopies during this study were similarly examined. Multivariate logistic regression analyses were used to establish odds ratios after controlling for potentially confounding variables.

## Results

Table 1 lists the results of the univariate analysis of 6087 colonoscopies that were evaluated in this study. Colonoscopies (n = 3729) performed in the morning were compared to colonoscopies performed in the afternoon (n = 2358). The crude completion rate to the cecum was 95.0% in the morning group while the completion rate to the cecum was 93.6% in the afternoon group (p = 0.02; odds ratio [OR] 1.30, 95% confidence intervals [CI] 1.04–1.62).

**Table 1:** Univariate Anaylsis.

	AM	PM	p-value	
Mean Age (range)	63.3 (18–93)	64.0 (16–92)	0.31	
Male	2215 (59.4%)	1514 (56.9%)	0.06	
Female	1342 (40.6%)	1016 (43.1%)	0.06	
GI Fellow	318 (8.5%)	216 (9.2%)	0.39	
PEG Bowel Prep	3593 (96.4%)	2293 (97.2%)	0.07	
Reached Cecum	3544 (95.0%)	2208 (93.6%)	0.02	OR 1.30 (95% CI 1.04–1.62)
Acceptable Bowel Prep	3520 (94.5%)	2114 (90.2%)	<0.001	OR 1.86 (95% CI 1.53 – 2.25)

Mean Age reported in years. GI Fellow defined as involvement of GI fellow during all or a portion of the examination. PEG Bowel Prep defined as polyethylene glycol electrolyte solution bowel preparation. Acceptable Bowel Prep indicates bowel preparation quality designated excellent; good; or fair, adequate exam. OR: odds ratio. CI: confidence intervals.

The indication(s) for the examinations are listed in Table 2. Bowel preparation quality was assessed in the two groups. Overall, the quality of the bowel preparation was superior in the AM group as indicated by higher proportions of "excellent," and "good," and lower proportions of "fair, adequate exam," fair, exam compromised," and "poor." Acceptable bowel preparations (excellent; good; fair, adequate exam) were observed in 94.5% of patients in the AM group versus 90.2% in the PM group (p < 0.001; OR 1.86, 95% CI 1.53–2.25) (Table 1).

**Table 2:** Indication(s) for Examinations.

	AM	PM
Screening/Surveillance		
Screening/Average risk	1687 (45.2%)	937 (39.7%)
Surveillance Adenomatous Polyps	669 (17.9%)	390 (16.6%)
Screening/(+) Family History	299 (8.0%)	153 (6.5%)
Diagnostic		
Hematochezia	257 (6.9%)	207 (8.8%)
Diarrhea	239 (6.4%)	187 (7.9%)
Abdominal Pain	182 (4.9%)	140 (5.9%)
Constipation	93 (2.5%)	80 (3.4%)
Change in Bowel Habits	97 (2.6 %)	78 (2.6%)
Anemia	92 (2.5%)	66 (2.8%)

All indications >2.0% in each group. Multiple indications are given for some procedures, thus percentages do not exactly equal 100%.

Binary logistic regression was used to assess predictors of success or failure of cecal intubation for each of the independent variables listed above and to further understand the impact of the covariate control variables. Data was excluded from 146 examinations because they did not receive an appropriate bowel preparation quality designation. Logistic regression revealed three independent variables that impacted cecal intubation rates (Table 3). Male gender was significantly associated with higher (OR 1.61, 95% CI 1.28 – 2.02) completion rates while, conversely, female gender was significantly associated with lower completion rates. Increasing age was associated with a significantly lower completion rate (OR 0.98, 95% CI 0.97 – 0.99). Acceptable bowel preparation was associated with higher cecal intubation rates compared to unacceptable bowel preparation (OR 5.43, 95% CI 4.13 – 7.14). No other variables, including involvement of gastroenterology fellow, type of bowel preparation, individual endoscopist or indication(s) impacted completion rates. After controlling for these variables, no significant difference was noted in cecal intubation rates in the morning versus afternoon. These data suggest cecal intubation rates are lower in the afternoon than in the morning and these differences can be accounted for by the effects of female gender, advancing age, and compromised bowel preparation.

**Table 3:** Multivariate Analysis.

Variable	OR	95% Confidence Interval	p-value
AM	1.11	0.88 – 1.39	0.37
Male Gender	1.61	1.28 – 2.02	<0.001
Age	0.98	0.97 – 0.99	0.001
Fellow	1.12	0.76 – 1.79	0.49
Acceptable Bowel Prep	5.43	4.13 – 7.14	<0.001

Multivariate logistic regression of colonoscopy completion rates, indicating the odds ration (OR) of each factor on completion rates. "AM" indicates colonoscopies begun prior to 12 noon. "Age" indicates increasing patient age. "Fellow" indicates involvement of gastroenterology fellow. "Acceptable Prep" indicates bowel preparation quality designated excellent; good; or fair, adequate exam.

## Discussion

The finding of lower unadjusted cecal intubation rates in the PM group has a number of implications. From an institutional perspective, the identification of such a discrepancy should lead to the exploration of methods to eliminate this difference. From a physician's perspective, afternoon examinations may be more challenging when bowel preparation quality is poorer and also less satisfying when the cecal intubation rate is lower. From a patient's perspective, morning examinations already seem to be preferable, in general. Our data suggest that morning examinations may be both more convenient and associated with a higher completion rate.

Adequate bowel preparation is a prerequisite for performing a quality colonoscopic examination. Generally, bowel preparations deemed "fair, exam compromised" and "poor" necessitate a repeat examination, and this is the reason for making the distinction between "acceptable" and "unacceptable" groups. Even without separating preparation quality into these two groups, significant differences are present in the categories assigned during the report between the morning and afternoon groups. This is most likely due to the patients beginning the bowel preparation twenty or more hours prior to the examination.

Physician and staff fatigue could be a factor in lower afternoon completion rates, but there is no evidence of this on multivariate analysis. If there had been no difference in any of the variables, we would have had to consider this possibility more strongly. Nevertheless, it is still possible that physician and staff fatigue is contributory, as the rating of bowel preparation quality is subjective and difficult to standardize. In our outpatient endoscopy unit 25 endoscopists practice and are routinely assigned half-day endoscopy calendars that vary between mornings and afternoons. Less than 5% of the time an individual endoscopist will perform colonoscopies in both the morning and afternoon on the same day. This practice pattern may or may not influence our results.

There are several limitations to our study. First, this data was collected retrospectively and is therefore subject to unforeseen confounding factors despite our best analysis. The CORI database includes a data entry field in which the reason for an incomplete examination should be listed. Omission of data in this field in 63% of the incomplete examination reports leaves our study without potentially corroborating information. If a high percentage of examinations suggested that the bowel preparation quality prevented the completion of the exam, then this would have been confirmatory. In the future we will consider making this field mandatory for completion of the report. Our practice patterns may not be universally applicable. For example, our patients took the bowel preparation the evening prior to the exam irrespective of the timing of colonoscopy on the following day. Additionally, other practices may have a significant number of procedures performed after a different bowel preparation regimen. The practice pattern of endoscopists rarely performing a full day of outpatient procedures may not be universally applicable, as well. Lastly, the presence or absence of diverticulosis was not examined in this study. The has been demonstrated to impact cecal intubation rates in at least one study. [7] For this study, that variable was not analyzed because while diverticulosis is routinely documented on the patient reports, it was not felt to be reliably documented in a searchable field.

Overall, our findings are similar to those reported by Sanaka, et al. [17] In both studies, cecal intubation rates were statistically lower in the afternoon and bowel preparation was statistically more impaired in the afternoon. However, there are several differences. First, age and gender were found to impact cecal intubation rates in our study which has been demonstrated in other studies. [2-10] Secondly, after correcting for the variables of gender, increasing age and impaired bowel preparation, there was no statistically difference cecal intubation rates between the morning and afternoon groups in our study. The larger number of patients in our study or differences in the patient populations or in the settings of the two studies may account for these findings.

A number of measures should be considered to address these findings. One method would be to alter the administration of the bowel preparation, particularly for afternoon exams, to consume all or a portion of the preparation on the same day of the colonoscopy. Splitting the 4L of PEG electrolyte solution into 2L consumed the evening prior to the exam and 2L consumed the morning of the exam has been shown to produce a higher quality bowel preparation compared to consuming 4L the evening prior to the procedure in one study. [18] Secondly, performing as many colonoscopies as possible in the morning (which would likely lead to more upper endoscopies in the afternoon) would improve completion rates based upon our data. Finally, selecting older patients for morning appointments may also improve quality. Each of these measures should be explored in a prospective fashion to formulate a strategy that optimizes bowel preparation quality.

## Conclusion

The uncorrected cecal intubation rate at our institution is lower for afternoon examinations compared to morning examinations, and bowel preparation quality is significantly worse in the afternoon compared with the morning. Female gender, increasing age and poorer bowel preparation quality were associated with significantly lower cecal intubation rates. After adjusting for these contributing variables, no difference in cecal intubation rates was noted in this study. Prospective studies are needed to evaluate methods to improve afternoon bowel preparation quality, to reduce the number of compromised examinations and to reduce the need for repeat examinations.

## Competing interests

The author(s) declare that they have no competing interests.

## Authors' contributions

CDW, RIH, VKS participated in the conception and design of the study. MDC, NM performed the statistical analysis. CDW, RIH, VKS, SRG, JAL, DEF helped to draft the manuscript. All authors read and approved the final manuscript.

## Pre-publication history

The pre-publication history for this paper can be accessed here:


